# The State of Gerontological Training in Ghana: Implications for a Specialized Workforce and the Aging Population

**DOI:** 10.1093/geroni/igab046.1353

**Published:** 2021-12-17

**Authors:** Samuel Asante, Grace Karikari

**Affiliations:** 1 Northeastern State University, Broken Arrow, Oklahoma, United States; 2 University of North Dakota, Grand Forks, North Dakota, United States

## Abstract

The rise in older population in Ghana is accompanied by several challenges that may require trained professionals with specialized knowledge in geriatrics and gerontology to help address. Extensive review of existing literature, however, indicates a lack of comprehensive geriatric focused training in Ghana’s education system. Unfortunately, the scope of this training deficiency, potential impact on the geriatric workforce, as well as the health and wellbeing of the aging population on the national level have not been extensively examined. With the older adult population set to increase over the next few years, the need for geriatric-trained human service professionals in diverse disciplines, including medicine, nursing, psychology, social work and public health has become more pronounced. This paper is a review of the current state of gerontological education in higher institutions in Ghana. Specifically, authors examined (1) current geriatric-focused training programs available to students in all public/government owned institutions, (2) existing national level programs, and policies addressing training deficiencies, and (3) the implications of findings for future geriatric workforce as well as the health and wellbeing of older Ghanaians. The findings call attention to the need for a nationwide geriatric-focused training system and policies aimed at prioritizing specialized care for the older population. Culturally appropriate recommendations for integrating gerontological training and education in tertiary institutions are discussed. Guidelines and standards based on the Academy for Gerontology in Higher Education (AGHE) Competencies for Undergraduate and Graduate Education are proposed.

